# Diatoms as an indicator for tile drainage flow in a German lowland catchment

**DOI:** 10.1186/s12302-018-0133-5

**Published:** 2018-02-15

**Authors:** Naicheng Wu, Claas Faber, Uta Ulrich, Nicola Fohrer

**Affiliations:** 1 Department of Hydrology and Water Resources Management, Institute for Natural Resource Conservation, Kiel University, Kiel, Germany; 2 Aarhus Institute of Advanced Studies, Aarhus University, Høegh-Guldbergs Gade 6B, 8000 Aarhus C, Denmark

**Keywords:** Diatoms, Indicator value method, Runoff components, Tracer

## Abstract

**Background:**

The separation of runoff components within a model simulation is of great importance for a successful implementation of management measures. Diatoms could be a promising indicator for tile drainage flow due to their diverse preferences to different aquatic habitats. In this study, we collected diatom samples of 9 sites (4 tile drainage, TD, and 5 river sites, Ri) in a German lowland catchment at a weekly or biweekly time step from March to July 2013 with the aim of testing the suitability of diatoms for tile drainage flow, which is typical for lowland catchment.

**Results:**

*Planothidium lanceolatum*, *Ulnaria biceps*, and *Navicula gregaria* dominated in TD sites with relative abundances of 22.2, 21.5, and 10.9%, respectively. For Ri sites, the most abundant species was *Navicula lanceolata* (20.5%), followed by *Ulnaria biceps* (12.9%), *Cyclotella meneghiniana* (9.5%), and *Planothidium lanceolatum* (9.3%). Compared with Ri sites, TD had a lower diatom density, biomass, species richness, and percentage of Aquatic/Riparian diatoms (AqRi%). However, the proportion of Riparian diatoms (RiZo%) increased at TD. Indicator value method (IndVal) revealed that the two groups (Ri and TD) were characterized by different indicator species. Fifteen taxa, including *Cocconeis placentula*, *Cyclotella meneghiniana*, *N. lanceolata*, and *U. biceps*, were significant indicators for Ri sites. *Planothidium lanceolatum*, *Achnanthidium minutissimum*, and *Navicula gregaria* were significant indicators for TD sites.

**Conclusion:**

A pronounced variation was found in the species lists of diatom community between Ri and TD water body types associated with different indicator species. With respect to hydrograph separation, these findings highlight the suitability of diatoms as an indicator for tile drainage flow. However, spatial and temporal variations of diatoms should be considered in future surveys.

## Background

Lowland areas are characterized by low hydraulic gradients, shallow groundwater, flat topography, high potential for water retention, and a large amount of tile drainages in agricultural areas [1–4]. In such regions, drainage flow plays an important role and should be considered in modeling [5]. As a process-based ecohydrological river basin model, the SWAT model (Soil and Water Assessment Tool) [6] has already been used successfully in lowland catchments [1, 7–9]. However, the influence of drainage networks, groundwater dynamics, wetlands, and ponds on model performance was very pronounced [4]. Besides, tile drainages are difficult to incorporate into models using standard data sources and techniques due to the lack of information about the location and characteristics of the tile drainage system [1]. For a successful implementation of management measures, the separation of runoff components within a model simulation becomes of particular interest, when the transport pattern of contaminants (e.g., phosphorus, nitrogen, pesticide) is the target of a model application. In this regard, hydrologists are endeavoring to search reliable tracers that can identify and assess runoff-generating processes and detect sources of stream flow components within a target catchment [10–12]. Common tracers may be stable isotopes (Deuterium, oxygen-18, etc.) and radioactive isotopes (i.e., carbon-14). Biotic tracers, which may be applied for runoff process studies, are algae. Diatoms, for example, have been identified by [13, 14] as a potential tracer for surface runoff. Recent studies have investigated the relationships between stream diatoms with hydrological variables [15] as well as terrestrial and riparian diatoms and found that the origin of diatom species during floods partly stemmed from riparian and/or terrestrial-upland habitats [14, 16]. Nevertheless, the use of diatoms as a tracer in catchment hydrology is still very limited up to now [14].

Diatoms are unicellular, eukaryotic algae with high species diversity, which can be observed in nearly every aquatic environment including fresh and marine waters, moist terrestrial habitats, such as soils, rock surfaces, or epiphytes [17, 18]. A distinct feature of diatoms is their highly differentiated cell wall (called frustule), which mainly consists of silica (SiO_2_). Their frustules consist of two valves and show an enormous diversity in shape. These species-specific cell wall ornamentations enable the diagnosis of diatoms and form the basis of diatom taxonomy and systematic. Diatoms have been widely applied in marine ecosystem research [19–22] and have a high potential as tracers of particular matters or processes such as sources of suspended matter [23], coastal upwelling [24], and climate change [25]. In streams and rivers, diatoms are commonly used as reliable environmental indicators based on several merits, e.g., base of food webs and food chains [26], high sensitivity to physico-chemical and biological changes [27, 28], and cosmopolitan character with a wide geographical distribution and well-known autecology of most species [29, 30]. The small cell with sizes varying commonly between 10 and 200 µm in diameter or length [31] allows them to be easily transported by water. As a consequence, many assessment methods based on diatoms have been developed in several countries and regions [22, 32] for different environmental stressors such as flow regulation [33–35], nutrient enrichment [36], and heavy metal pollution [37].

Nevertheless, diatoms have so far not been introduced as general freshwater tracers of hydrological processes, especially in lowland areas. Based on the habitat preferences of distinct diatom species [29], a higher concentration of terrestrial diatoms is related to the occurrence of surface runoff during runoff events. Considering the potential usage of drift diatoms to link the terrestrial and aquatic worlds, Pfister et al. [13] proposed to use diatoms as a tracer of water resources and hydrological connectivity in the mountainous Attert catchment. The preliminary results of Martínez-Carreras et al. [38] showed that diatoms can help detect the onset/cessation of surface runoff. However, apart from the above-mentioned studies, investigations on diatoms as tracers are rarely found, and to our knowledge, except for [13, 14, 16], there is no investigation using diatoms as a tracer of hydrological processes.

The objectives of this study are to (1) investigate diatom assemblages at tile drainage sites, (2) compare the diatoms between river and tile drainage sites, and (3) identify indicator species for tile drainage sites in a German lowland catchment. Our hypothesis is that the diatoms at tile drainage sites are different from river sites due to their particular habitats.

## Methods

### Description of the study area

**Fig. 1:**
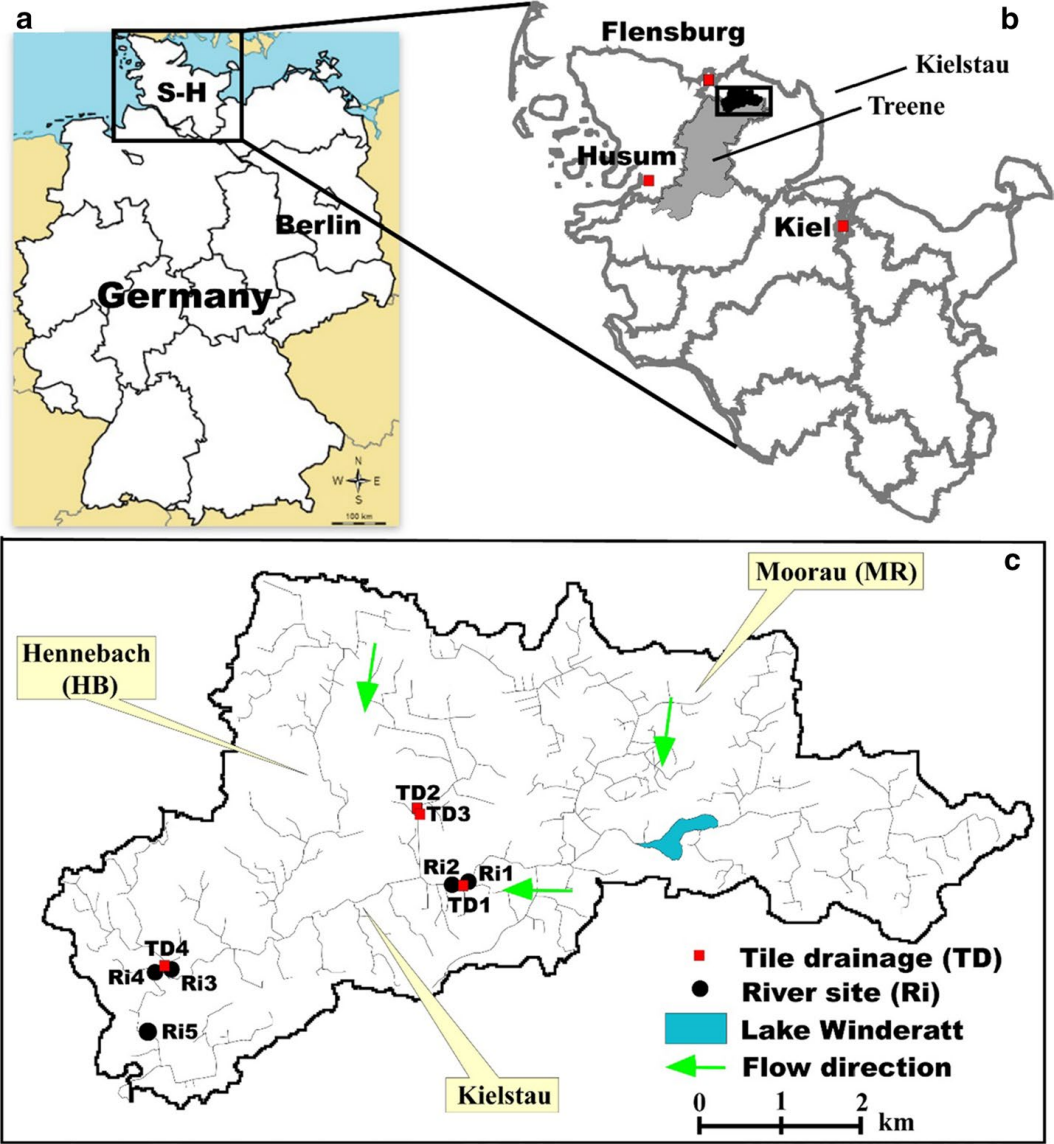
The location of sampling sites in Kielstau catchment (**c**), Schleswig–Holstein state (**b**), Northern Germany (**a**). S–H = Schleswig–Holstein state

The Kielstau catchment, an UNESCO demosite for Ecohydrological since 2010 [39], is a lowland watershed with a drainage area of 50 km^2^, and located in the Northern part of Germany (Schleswig–Holstein). It has its origin in the upper part of Lake Winderatt and is a tributary of the Treene River (Fig. 1) [27]. The precipitation is 841 mm/a (station Satrup, 1961–1990) [40] and the mean annual temperature is 8.2 °C (station Flensburg, 1961–1990) [40]. Moorau (MR) and Hennebach (HB) are two main tributaries within the Kielstau catchment. The drained fraction of agricultural area in the Kielstau catchment is estimated to be 38% [41] and various small tributaries and water from drainage pipes and ditches discharge into the river Kielstau. Sandy, loamy, and peat soils are characteristic for the catchment. Land use is dominated by arable land and pasture (~ 55 and ~ 26%, respectively, of the catchment area) [4, 41].

### Sampling methods and primary procedures

At 4 tile drainage (TD) and 5 river sites (Ri), samples were taken weekly or biweekly from March to July 2013. A total of 40 tile drainage and 63 river samples were analyzed. At each site and on every sampling date, the volume of diatom samples was determined and samples were filtered through a 20-µm plankton net. The retained organisms were transferred into 50-mL glass bottles and fixed in 5‰ non-acetic Lugol’s iodine solution [42]. After 48 h, the supernatant liquid of undisturbed samples is carefully removed and samples are thus concentrated to 30 mL for further processing.

Simultaneously, at each sampling point, water temperature (WT), pH, electric conductivity (EC), and dissolved oxygen (DO) of the surface water were measured in situ using Portable Meter (WTM Multi 340i and WTW Cond 330i, Germany). Discharge (m^3^/s) was calculated by either beaker with timer (for TD sites with small discharge such as TD1, TD2, and TD3) or velocity–area method at the sampling points (for stream sites and TD4) (velocity—using FlowSens Single Axis Electromagnetic Flow Meter, Hydrometrie, Germany). Concurrently, water samples were taken in two pre-cleaned plastic bottles (500 mL each) for water chemistry measurement in the laboratory. In the lab, water samples were partially filtrated through GF/F glass microfiber filter (Whatmann 1825-047) for measurements of phosphate-phosphorus (PO_4_-P), ammonium-nitrogen (NH_4_-N), nitrate-nitrogen (NO_3_-N), nitrite-nitrogen (NO_2_-N), chloride (Cl^−^) and sulfate (${\text{SO}}_{4}^{2 - }$) according to the standard methods DEV (Deutsche Einheitsverfahren zur Wasser-, Abwasser- und Schlammuntersuchung). The concentrations of total phosphorus (TP) were measured with unfiltered water samples. PO_4_-P and TP were measured using the ammonium molybdate spectrophotometric method (at 880 nm; DIN 1189). We used Nessler’s reagent colorimetric method (DIN 38 406-E5-1) to measure NH_4_-N concentrations at 690 nm. NO_3_-N, NO_2_-N, Cl^−^, and ${\text{SO}}_{4}^{2 - }$ were measured by an ion chromatography method (DIN 38 405-D19).

### Identification under microscope

Permanent diatom slides were prepared after oxidizing the organic material by nitric acid and sulfuric acid and a minimum of 300 valves were counted for each sample using a Zeiss Axioskop microscope at 1000× under oil immersion. Diatoms were identified to the lowest taxonomic level possible (mainly species level) according to Simonsen [43], Round et al. [17], and Lange-Bertalot [44–47]. Their densities were expressed as cell/L. Diatom biomass was estimated by taxa biovolumes (by closest geometric form supposing specific gravity of 1.00 g/cm^3^) [48, 49].

### Data analyses

Besides total density and total diatom biomass, we calculated community diversity indices. They were Berger–Parker diversity [50], evenness [51], HillN1 diversity [52], Margalef’s diversity [53], McNaughton diversity [54], Menhinick diversity [55], Shannon–Wiener diversity (H’) [56], Simpson’s Dominance, and species richness to describe the diatom assemblage. Since the occurrence of diatom species is bound to specific habitat and wetness conditions, diatoms were classified into five moisture categories and then we calculated their relative abundances [29]: Aquatic zone (AqZo%), Aquatic/Riparian transition zone (AqRi%), Riparian zone (RiZo%), Riparian/Upland transition zone (RiUp%), and Upland zone (UpZo%) (Table 1). The classification was also reorganized into two habitat categories, namely Aquatic% and Terrestrial%. Mann–Whitney U tests were used to compare their differences between Ri and TD sites.

**Table 1:** Classification of the moisture indices

Category	Code	Diatom habitat	Diatom occurrence
1	AqZo	Aquatic zone	Never, or only very rarely, occurring outside water bodies
2	AqRi	Aquatic/Riparian transition zone	Mainly occurring in water bodies, sometimes on wet places
3	RiZo	Riparian zone	Mainly occurring in water bodies, also rather regularly on wet and moist places
4	RiUp	Riparian/Upland transition zone	Mainly occurring on wet and moist or temporarily dry places
5	UpZo	Upland zone	Nearly exclusively occurring outside water bodies

The indicator value method (IndVal) was used to detect how strongly each species discriminated between Ri and TD groups. The indicator value of a taxon varied from 0 to 100, and the indicator value attained its maximum value when all individuals of a taxon occurred at all sites within a single group. We tested the significance of the indicator value for each species with a Monte Carlo randomization procedure with 1000 permutations. We ran IndVal with PC-ORD (Version 4; MjM Software Design, Gleneden Beach, Oregon).

## Results

**Fig. 2:**
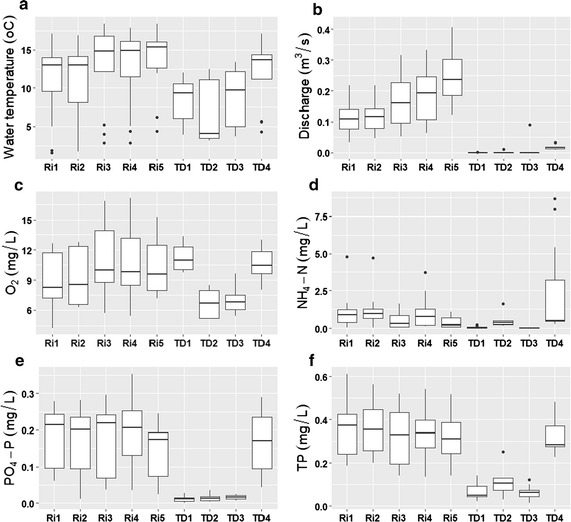
Box plots of main environmental conditions including water temperature (**a**), discharge (**b**), O_2_ (**c**), NH_4_-N (**d**), PO_4_-P (**e**), and TP (**f**) at different sampling sites. Boxes show interquartile ranges (25th and 75th%), middle lines are medians, whiskers are non-outlier ranges beyond the boxes, and dots are outliers

Study reaches varied greatly in water-quality and habitat characteristics (Fig. 2). In comparison to river sites (Ri), sites of tile drainages (TD) demonstrated a lower average temperature (Fig. 2a), O_2_ contents (except for TD1 and TD4) (Fig. 2c), and smaller discharge (Fig. 2b). Nutrient concentrations (e.g., NH_4_-N, PO_4_-P, and TP) of TD1–TD3 were dramatically lower than those of Ri sites and TD4 (Fig. 2d–f). For example, the mean discharges of TD1–TD4 were 0.21 L/s, 1.97 L/s, 9.04 L/s, and 18.05 L/s, respectively, while the discharges of Ri1–Ri5 averaged 85.39 L/s, 114.06 L/s, 168.06 L/s, 186.12 L/s, and 241.50 L/s, respectively. The TP concentrations of TD1–TD3 were 0.067 mg/L, 0.119 mg/L, and 0.062 mg/L, respectively. In contrast, TP concentrations of Ri1–Ri5 and TD4 were 0.365 mg/L, 0.368 mg/L, 0.325 mg/L, 0.326 mg/L, 0.311 mg/L, and 0.315 mg/L, respectively.

A total of 78 diatom species were recorded in this study. Within all samples, *Navicula lanceolata* (Ehrenberg), *Ulnaria biceps* (Kützing), and *Planothidium lanceolatum* (Brébisson ex Kützing) were the most abundant species, whose relative abundances were 17.7, 14.4, and 11.5% of the total abundance, respectively. *Planothidium lanceolatum*, *Ulnaria biceps*, and *Navicula gregaria* (Donkin) dominated in tile drainage (TD) sites with relative abundances of 22.2, 21.5, and 10.9%, respectively. For river (Ri) sites, the most abundant species was *Navicula lanceolata* (20.5%), followed by *Ulnaria biceps* (12.9%), *Cyclotella meneghiniana* (Kützing) (9.5%), and *Planothidium lanceolatum* (9.3%). In general, TD had lower diatom density, biomass, species richness, HillN1 diversity, and percentage of Aquatic/Riparian diatoms (AqRi%) than those of Ri (Table 2). However, the proportion of Riparian diatoms (RiZo%) increased at TD (Table 2).

**Table 2:** The comparisons of diatom indices between river (Ri) and tile drainage (TD) sites

Diatom indices	Ri (n = 63)				TD (n = 40)			
	Min	Max	Mean	SD	Min	Max	Mean	SD
Density (cells/L)**	*33,480*	*750,804*	*202,544*	*113,400*	*156*	*565,041*	*67,643*	*121,417*
Biomass (mg/L)**	*0.09*	*2.53*	*0.52*	*0.37*	*0.00*	*1.93*	*0.19*	*0.37*
Diversity indices								
Margalef’s**	*0.79*	*2.21*	*1.26*	*0.24*	*0.00*	*1.47*	*0.82*	*0.40*
Evenness	0.47	0.88	0.78	0.08	0.44	1.00	0.81	0.15
Richness**	*14.00*	*35.00*	*22.89*	*4.00*	*1.00*	*25.00*	*13.03*	*6.68*
Shannon–Wiener**	*1.56*	*2.87*	*2.42*	*0.29*	*0.00*	*2.73*	*1.84*	*0.65*
Simpson’s**	*0.08*	*0.47*	*0.15*	*0.07*	*0.09*	*1.00*	*0.26*	*0.21*
Berger–Parker*	*0.14*	*0.68*	*0.29*	*0.12*	*0.17*	*1.00*	*0.38*	*0.20*
McNaughton**	*0.27*	*0.72*	*0.42*	*0.11*	*0.30*	*1.00*	*0.56*	*0.20*
Odum**	*0.03*	*0.81*	*0.16*	*0.14*	*0.03*	*12.26*	*1.96*	*2.51*
Menhinick**	*0.03*	*0.17*	*0.06*	*0.02*	*0.02*	*0.37*	*0.11*	*0.07*
HillN1**	*4.76*	*17.61*	*11.70*	*3.10*	*1.00*	*15.31*	*7.38*	*3.64*
Moisture indices								
AqZo%	3.70	38.24	17.29	7.51	0.00	76.67	19.60	18.03
AqRi%**	*25.00*	*80.08*	*53.58*	*14.68*	*0.00*	*75.00*	*23.84*	*17.74*
RiZo%**	*7.85*	*63.33*	*28.13*	*12.16*	*0.00*	*100.00*	*52.26*	*23.61*
RiUp%	0.00	4.21	1.00	1.05	0.00	100.00	4.30	16.01
UpZo%	0.00	0.00	0.00	0.00	0.00	0.00	0.00	0.00
Aquatic%	95.79	100.00	99.00	1.05	0.00	100.00	95.70	16.01
Terrestrial%	0.00	4.21	1.00	1.05	0.00	100.00	4.30	16.01

*AqZo* Aquatic zone, *AqRi* Aquatic/Riparian transition zone, *RiZo* Riparian zone, *RiUp* Riparian/Upland transition zone, *UpZo* Upland zone

*p* > 0.05; * *p* *<* *0.05*; ** *p* *<* *0.001* (Mann–Whitney U tests)

**Fig. 3:**
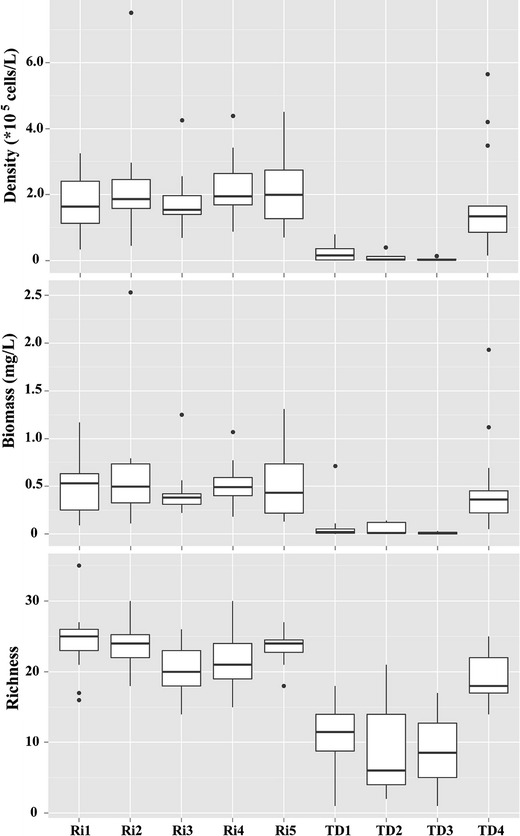
Box plots of diatom density, biomass, and species richness at different sampling sites. Boxes show interquartile ranges (25th and 75th%), middle lines are medians, whiskers are non-outlier ranges beyond the boxes, and dots are outliers. Other diversity indices performed similarly (not shown)

**Fig. 4:**
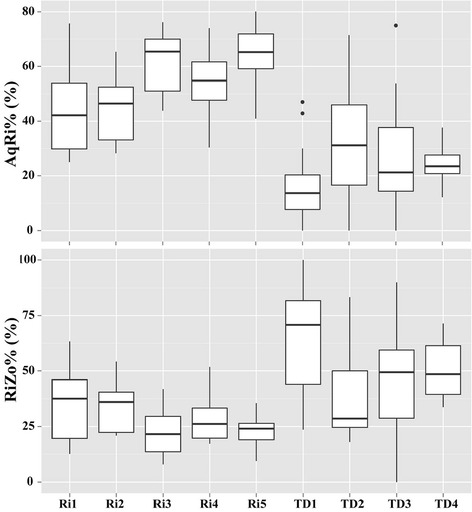
Box plots of AqRi% and RiZo% at different sampling sites. Boxes show interquartile ranges (25th and 75th%), middle lines are medians, whiskers are non-outlier ranges beyond the boxes, and dots are outliers. AqRi, Aquatic/Riparian transition zone; RiZo, Riparian zone

**Fig. 5:**
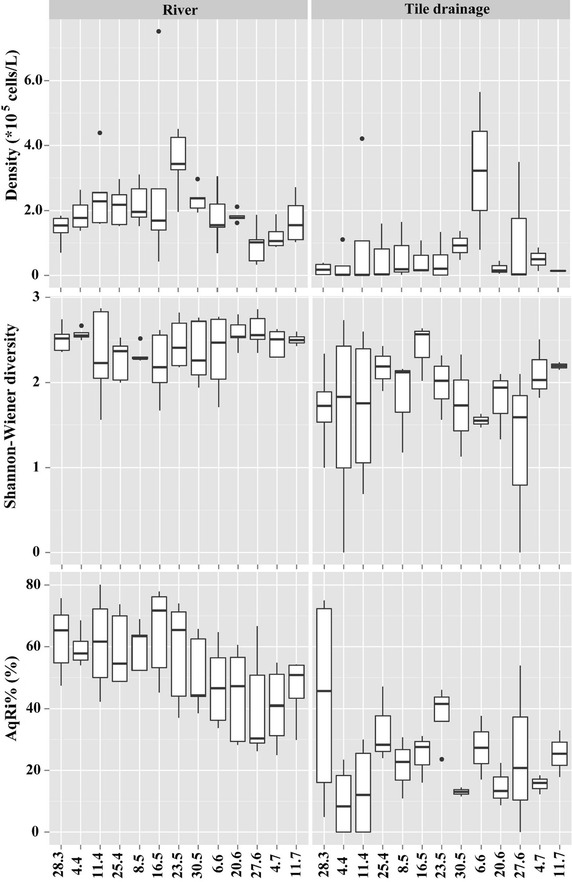
Temporal variations of different diatom indices at river (Ri) and tile drainage (TD) sites. Boxes show interquartile ranges (25th and 75th percentiles), middle lines are medians, whiskers are non-outlier ranges beyond the boxes, and dots are outliers. Other diversity indices performed similarly (not shown)

**Fig. 6:**
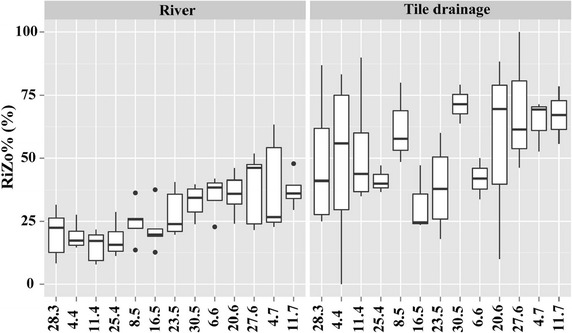
Temporal variations of RiZo% at river (Ri) and tile drainage (TD) sites. Boxes show interquartile ranges (25th and 75th percentiles), middle lines are medians, whiskers are non-outlier ranges beyond the boxes, and dots are outliers. Other diversity indices performed similarly (not shown)

Both diversity and moisture indices are in the same range at Ri sites (Ri1–Ri5) (Figs. 3, 4). Nevertheless, TD sites varied and TD4 exhibited a particular case. Similar with Ri sites, TD4 had higher diatom density, biomass, species richness than those of TD1–TD3 (Fig. 3). Whereas, moisture indices of TD4 (e.g., AqRi% and RiZo%) were quite similar with TD1–TD3 (Fig. 4). Temporal variations of different diatom indices are shown at Figs. 5, 6. Ri sites demonstrated clear temporal variations. For example, AqRi% decreased while RiZo% increased from March 28 to June 27, 2013. However, TD sites changed very randomly without a significant temporal trend as of Ri sites (Figs. 5, 6).

To identify the key indicator species of the river and tile drainage groups, the indicator value method (IndVal) was used and showed that the two groups were characterized by different indicator species (Table 3). Fifteen taxa, including *Cocconeis placentula* (Ehrenberg), *Cyclotella meneghiniana*, *Navicula lanceolata*, and *Ulnaria biceps*, were significant indicators for Ri sites. *Planothidium lanceolatum*, *Achnanthidium minutissimum* (Kützing), and *Navicula gregaria*, with small cell sizes, were significant indicators for TD sites.

**Table 3:** Summary of indicator species analysis showing indicator taxa, relative abundance, relative frequency, and indicator value (IV) for each group

Species	Relative abundance		Relative frequency		IV	
	Ri	TD	Ri	TD	Ri	TD
*Amphora ovalis* (Kützing)	95	5	27	3	*26*	0
*Cocconeis placentula* (Ehrenberg)	82	18	90	25	*74*	5
*Cyclotella meneghiniana* (Kützing)	74	26	92	55	*68*	15
*Fragilaria biceps* (Kützing)	61	39	100	60	*61*	24
*Fragilaria elliptica* (Schumann)	75	25	59	10	*44*	2
*Fragilaria leptostauron* (Ehrenberg)	100	0	14	0	*14*	0
	87	13	16	3	*14*	0
*Meridion circulare* (Greville)	61	39	83	48	*50*	19
*Navicula lanceolata* (Ehrenberg)	73	27	97	80	*71*	22
*Navicula perrotettii* (Grunow)	86	14	25	5	*22*	1
*Navicula viridula* (Kützing)	62	38	86	45	*53*	17
*Nitzschia sigma* (Kützing)	58	42	90	43	*52*	18
*Staurosira phoenicenteron* (Ehrenberg)	100	0	22	0	*22*	0
*Surirella elegans* (Ehrenberg)	63	37	68	25	*43*	9
*Synedra binodis* (Ehrenberg)	100	0	27	0	*27*	0
*Planothidium lanceolatum* (Brébisson ex Kützing)	32	68	100	90	32	*61*
*Achnanthidium minutissimum* (Kützing)	20	80	83	78	16	*62*
*Navicula gregaria* (Donkin)	33	67	92	85	30	*57*

*Ri* river sites, *TD* tile drainage sites

The italic numbers are significant indicator values (*p* < 0.05, Monte Carlo permutation test)

## Discussion

The analyses revealed a high variation in diatom community between tile drainage (TD) and river (Ri) sites in the study area with considerable different species composition and many species showed pronounced affinities with one water body type. For example, some species, like *Cocconeis placentula*, *Eunotia bilunaris* (Ehrenberg), *Fragilaria elliptica* (Schumann), *Melosira granulata* (Ehrenberg), *Synedra binodis* (Ehrenberg), and *Tabellaria flocculosa* (Kützing), were clearly associated with Ri sites. In contrast, *Navicula gregaria*, *Reimeria sinuata* (Kociolek & Stoermer) were mainly found in TD systems. *Cocconeis placentula* is relatively resistant to scour and prefers high current habitat because of the prostrate growth form and firm attachment via mucus secreted by the raphe valve. *Reimeria sinuata* with small cell size (8–20 µm) occurs mainly in water bodies, also rather regularly on wet and moist places [29]. Furthermore, compared with Ri sites, TD had much lower diatom density, biomass, and species richness (density: 0.68 × 10^5^ vs. 2.03 × 10^5^ cells/L, biomass: 0.19 vs. 0.52 mg/L, species richness: 13 vs. 23).

An important reason for these differences is probably the specific habitat character of tile drainage. Since the water flows through a subsurface drainage pipe, light is almost not available at TD sites. This significantly reduces the possibility of photosynthesis that absorbs sunlight to synthesize carbohydrates from CO_2_ and water. Therefore, theoretically diatoms should not exist at TD sites, but actually we did observe many diatom species. One hypothesis was that the detected diatoms at TD sites were transported through soil macropores during runoff events [57]. Our results supported this hypothesis since the dominant species of TD sites were either of small sizes (e.g., *Planothidium lanceolatum*, *Achnanthidium minutissimum*, and *Navicula gregaria*) or long filamentous cells (e.g., *Ulnaria biceps*). These characters allow them to be transported easily by flowing water. Nevertheless, on the other hand, if this hypothesis was right, the diatom species of TD sites should be categorized as terrestrial species (i.e., RiUp or UpZo) because they origin from the moist top soil. This was not the case in this survey since most diatoms of TD were AqRi and RiZo species (Figs. 5, 6). It is still not clear, how surface runoff and tile drainage flow interact with regard to runoff generation, dynamics of nutrient, and contaminant losses [58]. Further investigations are thus needed to clarify the source or transfer processes of the observed diatom species.

Regardless of the origins of TD diatoms, there may be several reasons why Ri sites are richer in species than TD sites: (1) in comparison with TD, rivers are more heterogeneous in space and are less susceptible to drying out. This allows a potentially higher number of species to successfully settle in these systems; (2) the degree of connectivity may also play an important role. Rivers tend to have larger catchment areas than tile drainage system and have therefore a higher chance of being colonized from neighboring water bodies; (3) local stress events (e.g., inflow of pesticides or nutrients) have a larger impact on small water bodies than on larger-sized systems.

A second finding of this study was that TD4 performed differently compared to the other 3 TD sites. This result is generally attributed to its catchment area and nutrient supply. TD4 has a larger catchment area than those of TD1–TD3, which could be judged by their discharges (Fig. 2). For example, on 28th March, the discharge of TD4 was 17 L/s contributing 9.05% of downstream river discharge, whereas the contribution of TD1 to downstream river was only 0.069% with a discharge of 0.071 L/s. Tile drainages with larger catchments have a greater physical habitat complexity. Therefore there is a higher possibility of gathering other sources of diatoms and being colonized from neighboring water bodies. Furthermore, there was a wastewater treatment plant discharging into TD4, which provided major nutrient inputs (i.e., nitrogen and phosphorus) for diatom growth. This implied that spatial variations of tile drainages were remarkable and should be noted in further investigations, especially with respect to hydrograph separation.

Temporal variations were observed at Ri sites, which was in accordance with a former study in the Kielstau catchment [59]. The importance of seasonality to many organisms in aquatic systems, including diatoms, is not yet well known. Microbiological fluxes are usually highest in summer as higher temperature causes higher biological activity and reproduction, particularly in humid environments when moisture is not a limiting factor [60]. However, in this study we did not detect significant temporal variations at TD sites (Figs. 5, 6). One explanation is the habitat character of tile drainage, as mentioned above, is relatively stable compared with river water and less influenced by short-term climate changes (e.g., temperature, sunshine, and wind changes). Furthermore, the groundwater influence in the tile drainages keeps the water temperature constantly cool. Moreover, the use of the plankton net with a mesh size of 20 µm inevitably results in the loss of species smaller than 20 µm (or in filament) and may have important consequences for the present results. Besides, the time span of this study is very short and tile drainage fell dry in summer when high temperatures could have an impact. Although we have found significant temporal variations of Ri sites, it should be noted that this study is based on a short-term sampling campaign during a low-flow period. The situation during floods may change and dramatically influence the results. It has particular significance when we aim to select tracers, which should be temporally constant or their variations should be known [61]. Thus, further studies will consist in assessing the temporal variations of diatom community for reducing uncertainties in hydrological process identification and quantification. In addition, a rain-event-based sampling campaign in a discharge dependent sampling mode is being conducted. These data could be used to detect hydrological processes and diatom community.

## Conclusion

We found a pronounced variation in the species lists of diatom community between Ri and TD water body types. Indicator value method (IndVal) revealed that 15 taxa and 3 taxa were significant indicators for Ri and TD sites, respectively. With respect to hydrograph separation, these findings highlight the suitability of diatoms as an indicator of tile drainage flow contribution. However, the source or transport pathways of the observed TD species need to be clarified. Spatial and temporal variations should be considered in a future survey.
